# How do Mental Health Services Foster Hope? Experience of People Accessing Services

**DOI:** 10.1007/s10597-022-01073-y

**Published:** 2023-01-07

**Authors:** Anne Honey, Nicola Hancock, Rebecca Barton, Bridget Berry, John Gilroy, Helen Glover, Monique Hines, Shifra Waks, Karen Wells

**Affiliations:** 1 Faculty of Medicine and Health, The University of Sydney, Camperdown, NSW Australia; 2 Enlightened Consultants, Brisbane, QLD Australia

**Keywords:** Hope, Recovery, Mental health services, Lived experience research

## Abstract

Hope is essential to mental health recovery, yet little is known about how mental health services can foster hope. This paper addresses the question: How can mental health services influence the sense of hope experienced by people who access their services? Sixty-one people who accessed a new mental health service were interviewed about their experiences, including about how the service had influenced their sense of hope. Interviews were analysed using constant comparative analysis. The data revealed that hope increased when people perceived positive changes in themselves and their circumstances: developing new understandings and perspectives; having effective strategies to manage challenges; seeing progress or having plans; and having support. Changes were attributed to three major features of the service: accessibility; staff competence and wisdom; and caring interactions. The findings highlight that, while individual clinicians are important, other interactions with services and the wider service context are also critical for facilitating hope.

## Introduction

Having hope is described as a central or core component of mental health recovery (Laranjeira & Querido, 2022; Leamy et al., 2011). People diagnosed with mental health issues have described it as intrinsic to life, sometimes hidden, and often more noticed in its absence than its presence (Murphy et al., 2022). Hope has been variously defined, with no universal agreement, and some have argued that there is limited utility in identifying a unified definition of hope because of its multifaceted and context-specific nature (Herrestad et al., 2014; McCormack et al., 2017). For the purposes of this paper, we define hope as “the feeling of having possibilities, trust in others and in the future, zest for life, expression of reasons and will to live, inner peace, optimism, associated with setting goals and mobilization of energy” (Querido, 2020, cited in Laranjeira & Querido, 2022).

Mental health service systems internationally are being expected to shift towards more recovery-oriented approaches to practice, where recovery is defined as “being able to create and live a meaningful and contributing life in a community of choice with or without the presence of mental health issues” (Australian Health Ministers' Advisory Council, 2013, p. 11). Thus, they are increasingly called on to be hope-inspiring and to promote or facilitate hope with those who seek their assistance (Australian Health Ministers' Advisory Council, 2013). Yet there is a dearth of literature delving into the specific experiences or practices that support people to find or reignite their sense of hope (Sælør et al. 2015a) especially from the perspectives of the people accessing the services. The lack of clarity or nuance regarding what recovery-oriented and hope-supporting practice looks like, or what it entails, has been identified as a barrier to the operationalisation of recovery principles and their translation into practice (Nugent et al., 2017).

Only recently have some researchers started to examine what experiences facilitate or support hope for people who access mental health services, and these are not specific to experiences of the actual services. Yeung et al. (2020), in what appears to be the most comprehensive exploration of hope-promoting experiences to date, analysed the experiences of hope reported in written accounts by 72 Australians accessing mental health services. Participants reported a range of hope-promoting experiences including both personal or internal experiences and experiences that involved interactions with other people, including families, friends, peers, community members, and mental health workers. Mental health workers were discussed most frequently in hope-promoting experiences of: knowing others believe in my ability to recover; feeling respected, listened to and believed; and feeling supported.

Another study (Sælør et al., 2015b; Sælør & Skatvedt, 2019) examined facilitators and barriers to hope experienced within the service system by people with both mental health and substance use problems in Norway. In terms of barriers, they identified themes of ‘battling bureaucracy’, ‘distance, disempowerment and de-individuation’ and ‘no clean slates’, however, these related primarily to welfare services, such as benefits, employment and housing, rather than the mental health system. Facilitators identified included help with practical challenges, trust, and authentic relationships.

Related research, while not specifically focused on hope, has also provided some information about the hope-supporting practices used by mental health workers. For example, in studies of desirable characteristics of health professionals, people who access services have described the following features as inspiring hope: collaborative working, treating them as equals, optimism, and belief in their ability to recover (Horgan et al., 2021; Wand et al., 2022). A recent meta-synthesis of Norwegian literature on recovery-oriented practices (Klevan et al., 2021) identified that to promote hope, practitioners needed to help people to believe in themselves and others and to help people to see and acknowledge future opportunities. However, most of the texts discussing hope that were included in this meta-synthesis involved perspectives of health professionals rather than the people accessing the services. Further, literature on recovery usually relates to people with long-term and severe mental health issues (McEvoy et al., 2012), thus limiting understanding of how hope is promoted across a broader spectrum of people who access mental health services.

Further exploration of the views of people who access mental health services about the contribution of those services to hope-promoting experiences will add to this emerging body of work. It will provide further guidance and insight to support practitioners striving to enhance their hope-promoting practice. As part of a state-wide evaluation of a new mental health service model in Victoria, Australia, people discussed the impact the service had on their sense of hope. This paper reports their insights, addressing the research question: How can mental health services influence the sense of hope experienced by people who access their services?

## Methods

### Study Design

This paper is a qualitative content analysis (Drisko & Maschi, 2015) of data drawn from in-depth interviews with people who accessed a new mental health service called HeadtoHelp. It uses data from a larger project, the aim of which was to understand people’s broader experience of accessing this service. The Human Research Ethics Committee at The University of Sydney approved the project (Project #2021/222). Lived experience informed the project design and it was conducted by a team of researchers independent of HeadtoHelp, including researchers with and without lived experience of mental health challenges. There are no known conflicts of interest. All authors certify responsibility for the manuscript.

### Context

HeadtoHelp is a new service model in which people contact mental health services via a single, central intake line. All Victorians, regardless of age, mental health history or even citizenship status are eligible to use HeadtoHelp services. After a standardized needs assessment, they are either referred to a suitable external service or provided with appropriate services at one of 15 HeadtoHelp locations across the state. Services are most frequently one-to-one talking therapies provided by psychologists, but clinical care coordination, psychosocial support and other services are also available from professionals such as nurse practitioners, occupational therapists, psychiatrists, and peer workers depending on the location. Services are commissioned by Primary Health Networks (organisations funded by the government to manage health regions) and delivered by non-government organisations. HeadtoHelp was initiated by the Australian government in response to increased demand for mental health services triggered by COVID-19 and the associated restrictions. HeadtoHelp was intended and found to address a gap in the mental health service system for people whose needs are too complex for treatment by a general practitioner or via the limited psychological treatments partially subsidised by the federal government, but not severe enough to access state- funded specialist mental health services (Nous Group & The University of Sydney, 2022). People who use the services are a diverse mix ranging from those who have never contacted a mental health service before to people experiencing long-term mental health issues. While most people present with symptoms rather than pre-existing diagnoses, the majority present with high or very high psychological distress and are assessed as requiring moderate-intensity services (Nous Group & The University of Sydney, 2022). Although initially funded for the year ending September 2021, HeadtoHelp has since been rolled out nationally as “Head to Health”.

### Sampling and Recruitment

The sample was purposively selected from 386 people who had accessed HeadtoHelp and who responded to a call for expressions of interest (EOIs). The invitation was sent via text message to a total of 3911 people who had completed a HeadtoHelp intake assessment between September 2020 and August 2021. They were sent by HeadtoHelp, and participants responded to the independent researchers directly, ensuring that the researchers did not have contact details from non-volunteers and HeadtoHelp did not know who volunteered.

Maximum variation sampling (Palinkas et al., 2015; Patton, 2001) was used to obtain sample diversity across a range of dimensions (from information provided in the EOI) such as gender, age, cultural background, area, and level of service satisfaction. People selected were provided with study information verbally and via an information sheet. Those who agreed to participate provided informed consent and were subsequently interviewed. Further sampling focused on filling gaps in diversity based on existing participants. Sampling continued until subsequent interviews were not adding to the coding framework and categories were well described. A total of 90 people were contacted, of whom 61 consented to participate.

### Data Collection

In-depth, semi-structured interviews (Rubin & Rubin, 2004) were conducted by teleconference or telephone, depending on participant preference, and primarily by the lived experience researchers on the team. An interview guide containing broad, open-ended questions was used flexibly to help participants describe their experiences of interacting with HeadtoHelp. While the theme of hope was often mentioned by participants in the context of general questions about the impact of HeadtoHelp on their lives, people were also asked a specific question toward the end of the interview: “Did your experience with HeadtoHelp influence your sense of hope or having a positive future?” Depending on people’s responses they were asked to elaborate on the difference made and what experiences or features of the service they attributed the difference to. Interviews lasted from 16 to 143 min in total (mean = 40 min) and were audio-recorded and transcribed with participants’ permission.

### Data Analysis

Data were analysed using constant comparative analysis, a systematic and well-regarded qualitative analysis method associated with Grounded Theory (Charmaz, 2014; Glaser, 1978), but equally useful for content analysis coding. This involves line-by-line coding of transcripts, where each chunk of data is examined to identify underlying concepts which are named and become the codes. Each new chunk of data is compared to previous data and existing codes to determine underlying similarities and differences, and thus the need for new codes. Comparing codes to each other results in the grouping of codes into higher level categories defining how these relate to each other. Coding was conducted by three members of the research team, including two lived experience researchers. To facilitate rigour, all three coded the first two interviews, compared codes and came to a consensus. Ongoing discussions within the research team and comparison of interpretations, codes and categories helped to ensure that the analysis faithfully represented the views expressed by participants. Data were collected and analysed concurrently, with each interview being analysed as soon as possible afterward. This allowed the emerging codes and ideas to be explored in subsequent interviews. QSR NVivo was used to facilitate data management and coding. For this paper, codes relating to the concept of hope were extracted and explored in detail.

## Results

Participants were 61 people who had contacted HeadtoHelp between September 2020 and August 2021. Their characteristics are presented in Table 1. Quotations are identified by participant numbers.

**Table 1:** Participant characteristics

Characteristic	(n = 61)
Age: mean	43.0 years
**Age group**	
Under 18 years	1
18–24 years	8
25–34 years	13
35–44 years	13
45–54 years	13
55–64 years	4
65 + years	9
**Gender**	
Female	36
Male	19
Gender non-binary	6
**Diversity**	
Aboriginal or Torres Strait Islander background	9
Speaks a language other than English at home	12
Identifies with a culture other than “Australian”	21
Identifies as part of the LGBTQI + community	15
**Previous contact with mental health services**	
First time approaching mental health services	23
Previously used mental health services	37
**Area of residence**	
Melbourne	31
A regional city (e.g., Geelong, Mildura)	13
A rural area	16
Homeless	1
**Service journey after initial approach**	
No service	7
Counselling support or advice at initial call only	3
Referral to external service only	10
Service at HeadtoHelp	41
Service continuing at time of interview	26
Service completed at time of interview	15
**Would use HeadtoHelp again**	
Yes	46
No	11
Unclear	4

Of the 61 people interviewed, 44 said that interacting with the service had positively influenced their sense of hope. They described having a new or stronger belief that their life could get better or that they could manage their situation and did not need to fear the future. Four reported that this change was profound enough that they no longer considered ending their lives. As P50 said: “Honestly, I don’t think I’d be here without HeadtoHelp. It was getting to the point where I was giving up and it just—yeah, I was very suicidal.”

**Fig. 1:**
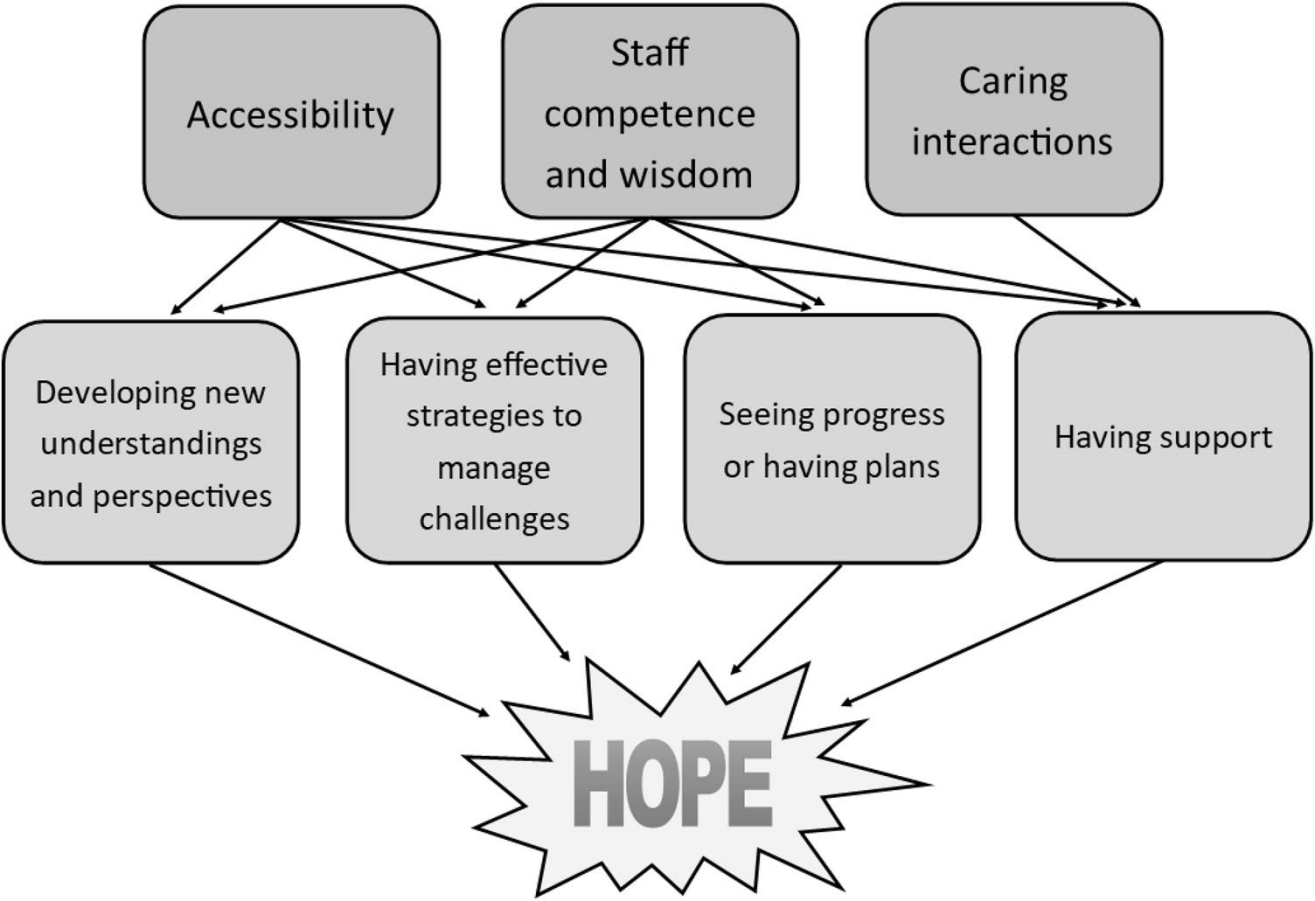
Pathways to increased hope from accessing a mental health service

People indicated that an increased sense of hope stemmed from their experiences of change in themselves and their circumstances: developing new understandings and perspectives; having effective strategies to manage challenges; seeing progress or having plans; and having support. These were closely entwined, with most people referencing experiences that involved changes in multiple areas. The changes, and thus hope, were attributed to three major features of the mental health service: accessibility; staff competence and wisdom; and caring interactions. Disappointment and reduced hope were experienced when these features were notably absent. These themes are depicted visually in Fig. 1 and elaborated in the following sections.

### Hope-Promoting Changes in Self and Circumstances

#### Developing New Understandings and Perspectives (n = *9)*

Nearly half of the participants who experienced an increase in their sense of hope attributed it, at least partly, to an increased sense of clarity or understanding of their own situation. This included people seeing their situations from a new perspective.

I was just so genuinely hopeless. I did not see the point of living, because I just am carrying all that baggage and not knowing why. When I actually got the chance to talk about things with [the counsellor], it completely shifted my point of view. I was like, okay. Maybe there is a point. Maybe it's not so bad (P8).

For some people, this new perspective included recognising their own agency and potential to change things for themselves.

I think they've just given me sort of a fresh look on things. They've given me hope that it is going to be okay. It's not, you know, you can work through things, it's not just done and dusted and that's it, it's all over. They've shown that, it's not going to happen overnight, but you can work through different things and life can get better (P14).

Others talked about gaining a more positive sense of self and self-worth. When asked about any change to their sense of hope, P10 responded that: “I believe that I’m worthy of a better life and that I deserve better and I, internally I really believe that.” They explained that before engaging with the service “I was not believing that I deserved a better job, a better relationship and that I deserved respect and love. I wasn’t believing in it for some reason, and I think now I do”.

#### Having Effective Strategies to Manage Challenges (n = 18)

Many people described their sense of hope as emanating from gaining strategies to manage the challenges that they experienced or knew might recur in their lives. This gave them the confidence that they could improve their well-being and successfully deal with future setbacks or problems. P39 reported that: “I can build my own confidence… When I do get very low and depressed, I just have better ways of coping and dealing with things”. P49 similarly explained:

I feel very buffered against potential stresses I'm going to have in the future… It feels like I've got this little kit, yeah, to access when I need it… so I feel very hopeful about the future, which is very much a shift from when I, at the point of intake or when I'd first decided to access the service (P49).

These strategies were diverse, including cognitive strategies, self-care strategies, stress and distress management, communication and assertiveness strategies, managing relationships, involvement in meaningful activities, and the ability and willingness to reach out for professional support when needed.

#### Seeing Progress or Having Plans (n = 20)

A critical contributor to hope was when people could see evidence that things were starting to improve. Many reported that they now had better mental health and increased coping abilities. For example, P59 said “It just made me want to keep going purely because I am seeing the improvements within myself and within—I'm not where I was six, seven months ago”. Others, like P20, talked about improvements that they had been able to make to their life situations, which made the future look brighter: “I’m actually improving my life now. I’m not just kind of stagnating… It’s a gradual uphill slope”.

Some people who had not yet observed changes in themselves or their situation still reported feeling more hopeful because HeadtoHelp had helped them develop goals and a plan of how to get there. P53 described that: “There’s a new plan now that’s written up…. We’ve come up with a plan … I’m happy with where it’s headed. It’s in the right direction”.

#### Having Support (n = 22)

Many participants said that they felt more hopeful because they now believed that they were getting the mental health support they needed. Even people who had not been with HeadtoHelp for very long described feeling hopeful because, based on their initial interactions, they felt that HeadtoHelp would be able to provide them with needed support. P50 described this as: “I feel like the more I speak to them, the more I’m going to be able to progress and go forward.” Similarly, P51 said: “I think [HeadtoHelp] can influence some really positive behavioural changes or take me out of the circles … they can break that negative thinking… I think they will be really important on that journey”.

Some people, like P23, discussed how, while they were no longer receiving support from HeadtoHelp or had received a referral only, their sense of hope was improved by simply knowing that HeadtoHelp was there for them should they need it in the future. “I do know that there’s help now…I know that’s there… I don’t think about doing the worst of the worst and ending it or anything anymore”.

#### Decreased Hope

Not everybody’s experiences, however, resulted in increased hope. To obtain diversity, participants who were unsatisfied with HeadtoHelp were oversampled, so it should be remembered that the number of people who reported a neutral or negative impact on hope is not representative overall. Thirteen people reported some negative impact of their interaction with the service on their sense of hope, including two who were ambivalent, describing ways in which their hope had both increased and decreased. A further six people reported that the service had had no impact on their hopefulness. Decreased hope resulted when people were unable to get the assistance they needed from HeadtoHelp. P45, for example, had asked HeadtoHelp for a counselling referral and was told “they don’t have any there because they’re full, so you have to go, you know, like get a mental health plan… but I don’t have the resources or the money to pay for a counsellor.” This made them feel that “nothing will change, yeah, it’s just a sad state of affairs that we live in.” Being unable to get the support they needed made P7 “feel, like, more alone and more, like there was just hopelessness that I wouldn’t, there was something wrong with me that either, more they’d know that I wouldn’t be able to find somebody to help me.”

### Hope-Promoting Service Features

Participants described three major types of service features that they felt contributed to experiences of developing new understandings and perspectives, having effective strategies to manage challenges, seeing progress/ having plans, and having support. These were: accessibility; staff competence and wisdom; and caring interactions.

#### Accessibility

It almost goes without saying that each of the hope-promoting experiences depended on the person’s ability to access the service when they needed it. The service was made accessible to them by being cost-free and responsive, and having service duration determined by need.

For a number of people, the fact that HeadtoHelp was *cost-free* made the difference between getting help or not getting help, as they did not have the resources to pay for services. For others it allowed them to have enough appointments or reduced the stress on them and on their families that would have been caused by the additional financial burden required for private services. P59 explained that if it wasn’t for HeadtoHelp “I wouldn't know where else to go because a lot of [services] cost money and being at uni and living by myself… I can't afford that” (P59).

*Responsiveness* was extremely important and included several aspects. Many people (n = 42) talked about starting services within a short time of first contacting the service—often within a couple of weeks. Frequently, people explained that the alternative was to be put on a waitlist for a psychologist or the community mental health team and wait for several months. P33 said “They were back to me within two weeks for an appointment. It didn't take long… if [they had not contacted me within] a few weeks I kind of would lose perhaps confidence in the service”.

Responsiveness also meant that, once in the system, people felt they could contact HeadtoHelp and speak to someone relatively quickly and that they could recontact HeadtoHelp if a referral did not work out or if they needed help again after ceasing treatment, which they found reassuring. One of the effective strategies people reported having developed to manage challenges—reaching out for help when needed—specifically depended on this belief that support was readily available:

Just the reassurance to know how easy it is to link in. Like, it gives me peace of mind that, if things sort of like, the symptoms sort of come back and they are like, sort of uncontrollable, I'm not going to have to wait a long time to see someone (P25).

A number of people mentioned that it was very important that there was *no set limit* on the services that they could receive. This was often contrasted with the subsidised occasions of services covered under the government’s Mental Health Plan, which were often seen as insufficient to deal with complex issues. The lack of a fixed time limit made people feel that they could receive enough help to address their issues rather than feeling pressured to deal with problems quickly, leaving work undone, or having to start again with a new service.

It's ongoing and it's kind of until you want to stop or feel like you can stop rather than it being like a set amount of time to visit, and then you have to get your stuff sorted in those amount of times… I would still be in a really dark place if I hadn't had access to the free services so frequently (P38).

A couple of people described how the *very existence* of the service gave them hope that the mental health system was improving, thus leading to increased possibilities for future assistance for themselves and others.

Just the fact that it's a new service that’s better than what - or something new and additional to what's been there in the past I think gives you hope. There's a lot of changes happening and I do think they are heading in the right direction. But, so that is positive (P26).

As noted above, however, *help was found to be inaccessible* by some, affecting their hope in a negative way. Some were told that they were ineligible to receive a service from HeadtoHelp or referred to external services that were inappropriate, that they already knew about, that were unaffordable, and/or that had long waiting times. In some locations, people were told that HeadtoHelp itself had a waiting list of up to two months. The inability to access the help they needed was experienced as reducing their hope that they would ever find the help they needed and sometimes their willingness to continue to try to seek help for their problems. For P23: “After I got referred two or three times, that’s where I feel like there’s no more help anymore”. P19 reported that not being able to access needed support “did influence my ability to keep seeking help” and that “every knock back has made me wonder why bother, you know… so it's always quite hard for me to see a future at the moment.”

#### Staff Competence and Wisdom

Aspects like seeing progress, having effective strategies to manage challenges, and developing new understandings and perspectives were usually attributed to the competence and usefulness of the psychological counselling and advice provided by HeadtoHelp.

The experience of counselling, or specifically the right counselling, is more like it just gives you the tools to deal with the same problems you've been having, but better than you could have before … some of the therapies we went through together really helped with some issues (P60).

In other cases, the clinical service was not experienced as useful, calling into question the competence or suitability of the staff. P47 reflected “I remember looking forward to the calls, and then afterwards feeling a little bit deflated, like okay, is that all there is to it? … It wasn’t adequate”.

#### Caring Interactions

It was not just wise advice, strategies, and specific techniques that built hope. It was also having interactions with staff that communicated genuine care. This included not only the interactions with the clinician appointed to them, but the person (usually described as a nurse) who took their call, did their initial assessment, contacted them about their appointments or called to touch base on how they were going. Interactions that were authentic, encouraging and focused on what was important to them made people feel that they were, and would continue to be supported. P6, for example, noted that: “the nurse phone calls have made me feel like there’s somebody out there that gives a shit that I’m okay”. P36 remembered how “HeadtoHelp did not make me feel like I was wrong…it was quite relieving. It's like ‘oh my god, someone is actually being supportive and not dismissive’”.

Service efficiency and reliability was part of what made people feel cared for and supported. This included appointments being kept, people being reminded about upcoming appointments and followed up if they missed an appointment, and efforts being made to find appointment times that were convenient to people’s lives. In some cases, people were proactively followed up to ensure that they were engaged with the service. For example, P42 reported that “they reminded me that they didn't forget about me… [With] some services, their clients will fall by the wayside.”

In contrast, however, some people experienced a lack of caring and willingness to help them, expressed through things like calls not being returned, promised contacts not happening or a dismissive attitude. These experiences resulted in lack of hopefulness. P7, for example, said: “I was trying to tell them stuff and they’d cut me off… I didn’t benefit from [it], if anything they just really agitated me and made me feel worse and more hopeless about the situation.” P45 experienced the whole system as indifferent to her needs, saying “I’ve never had to be in the public health system like this. So I understand now why people are just helpless and why they actually do feel sad, because there is nothing they can do”.

## Discussion

While the different meanings and manifestations of hope amongst people living with mental health issues are increasingly being explored (e.g., McCormack et al., 2017; Murphy et al., 2022), less is currently known about the specific ways in which mental health services can promote or hinder the development of hope. This paper sought individuals’ qualitative perspectives on how a specific mental health service influenced their sense of hope, enabling concrete responses grounded in recent experiences. Unlike many studies, it also included people with a diverse history of service use, rather than focusing only on those with long-term issues and long-term service use. Using data collected from 61 people who contacted a new mental health service in Victoria, Australia, we found that a range of mental health service features promoted hope, which were classified into accessibility, staff competence and wisdom, and caring interactions. Our findings also illuminate the mechanisms by which services can promote hope—through assisting people to: understand their situations more clearly and develop new perspectives; acquire effective strategies to manage challenges; recognise their progress; develop future-focused plans, and by providing a reliable, supportive ‘safety net’ where tailored services can be accessed when needed.

The findings support previous research on recovery-oriented practice, which has emphasised the importance of mental health workers in promoting hope, particularly through caring interactions, such as: expressing genuine hope for the person’s recovery; pointing out future possibilities; treating the person as an individual; being authentic rather than keeping a ‘professional distance’; trusting the person; genuinely listening, belief and empathy (Horgan et al., 2021; Klevan et al., 2021; Sælør et al., 2015b; Schrank et al., 2012; Yeung et al., 2020). The importance of clinical competence has also been discussed (Schrank et al., 2012; Yeung et al., 2020), though less frequently. It is possible that the hope promoting features of effective treatment have been taken for granted.

However, this paper emphasises that promoting hope is not just the remit of individual recovery-oriented clinicians and their individual relationships with those who access their services. The wider context and service landscape can also either promote or prevent experiences of hope. First and foremost, services need to be accessible. This is a systemic funding issue requiring adequate recurrent investment to ensure that people experiencing mental health challenges can feel confident that they will be able to access appropriate services when and where they are needed. Experiencing long delays, rejections, limited or inadequate services, and bureaucratic barriers are not only devastating to people in need, but are antithetical to hope even when people do not currently require such services (Klevan et al., 2021; Sælør & Skatvedt, 2019). This is likely to impact long term recovery (Schrank et al., 2012).

We found that people experience hope based on a belief that support is available, competent and likely to be helpful. Yet the wider evaluation pointed out a need for better promotion of the service as many participants reported finding out about it serendipitously (Nous Group & The University of Sydney, 2022). Ensuring that pathways to mental health support are well known is an important part of accessibility and, therefore, hope at a broader level.

Not being limited in the number of service engagements was stressed as important for hope. It can empower people to engage in decisions around preparing for and ending treatment and ensures that these decisions are based on needs and goal achievement rather than bureaucratically imposed. Our findings suggest that to provide hope, community-based mental health services need to be funded at a level that enables people to be supported for the length of time they need, without this impacting upon the wait-list times for others seeking support. This is important if services are to be considered hope-promoting for the whole of population and not just for those few individuals who can gain access to them. Service designs that focus on developing effective and transferrable strategies that facilitate agency and support people to feel confident to leave the service may be less likely to succumb to oversubscription than might be feared by proponents of limited services.

Difficulties with recruiting and maintaining high quality staff, and lack of appropriate local services to refer to have been highlighted as issues in our recent service evaluation (Nous Group & The University of Sydney, 2022), and are likely reflected in some of participants’ reports of negative experiences. This attests to the need for system-wide reform. No service exists in a vacuum, and the availability and integration of other mental health services is a critical issue yet to be adequately addressed in the Australian mental health system (Council of Australian Governments Health Council, 2018).

Finally, within individual health services, a positive, caring, person-centred and recovery-focused design and orientation needs to be experienced, not only in people’s interactions with individual clinicians, but through all aspects of service engagement. This includes booking systems, follow-up, and interactions with all service staff. These findings mirror the hope-diminishing experiences reported by people with both mental health and substance issues with the social welfare system (Sælør & Skatvedt, 2019). These Norwegian participants described having to battle bureaucracy, long waiting times, broken promises for support, difficulty making contact with people who could help them, not being treated as individuals, and lack of definitive information about the services and their own status with them. As noted in Klevan et al.’s review, “flexibility and non-bureaucracy in services are also considered to be pivotal by service users, as experiencing generosity and being treated as an individual with unique needs and resources may enhance hope as part of recovery processes” (2021, p.9).

### Limitations

This study has a number of limitations. Participants had accessed a new state-wide service model in Victoria, Australia. While this allowed the discussion of hope to focus on a specific and concrete situation, readers will need to assess the applicability of the findings in their own cultural and service contexts. As with any study relying on voluntary participation, those who agreed to be interviewed may have had more positive or more intense experiences with the service than those who did not. While our sample was diverse, all participants spoke English well enough not to need an interpreter (although one was available). This fits with informal information received from services that few clients required interpreters, but means that the views of some people from diverse cultural and linguistic backgrounds were not captured. Recruitment invitations excluded people who were assessed by HeadtoHelp as needing acute services, people who were already accessing sufficient mental health support elsewhere, and those who had opted out of the mental health system entirely; thus their perspectives are also missing from this analysis. Interviews focused on people’s broad experiences with the service, limiting the length and depth of discussion possible about hope. However, the broader focus was also a strength as it did not limit participants to people invested enough in the concept of hope to volunteer for a specific study about it. This study did not allow for follow-up of participants to investigate whether the impact on hope was sustained. While this research contributes to addressing the gap in knowledge about how mental health services can influence a sense of hope, further research in different cultural and service contexts, with non-English speakers and with follow-up over time will further add to knowledge in this area.

## Conclusion

This study demonstrates the potential for a mental health service to foster hope in people who access it. It reinforces the importance of a skilled and outcomes-focused clinical workforce whose interactions with people accessing services is caring, empathetic and authentic. It also highlights: (1) the importance of other interactions with the service, such as welcoming administrative staff, easy contact, efficient and flexible booking systems, needs-based service duration and proactive follow-up; and (2) the crucial role of wider mental health systems in promoting hope, including the availability of sufficient cost-free or affordable services that are accessible to those who need them when they are needed.

## Data Availability

Data is potentially identifiable, therefore cannot be made freely available.
